# Book Review

**Published:** 1898-09

**Authors:** 


**Human Nature: its Principles, and the Principles of Physiognomy.** By Physicist. Part I. Pp. 128. London: J. & A. Churchill. 1897.—"Human natur'," to slightly modify a saying of the immortal Mr. Squeers, "is more easier imagined than described," and after reading this little book we are inclined to agree with him; for although the author modestly observes "our goal is to understand Human Nature," it is quite certain that no light is thrown on the subject by "Physicist." In fact, it is very difficult to understand the involved and complicated sentences and the strange notions that abound; one sample will be enough to illustrate this: "Notwithstanding the difference in chemical composition between the colouring matters of plants, animals, and aniline, the uniform appearance of yellow accompanying a definite condition, viz., a basic, and the absence or deficiency of oxygen or acid, indicates, it would seem, a broad principle as definitely as the colour sensations of red and blue."

**Radiography in Marine Zoology. The British Echinodermata.** By R. Norris Wolfenden, M.D. London: The Rebman Publishing Co., Ltd. 1897.—The application of the Röntgen ray to the skeletal structures of invertebrate animals is still in its infancy. Dr. Wolfenden has in this work produced a number of radiograms of British echinodermata, and has introduced for comparison photographs of the organisms in their natural state.

Although the results are not yet all that could be desired they are full of promise, and the process is likely to be of distinct value to the zoologist. No doubt if we take the common forms dissection is preferable. But radiography is not to supersede, but to supplement dissection. And there are many forms which are not common, but exceedingly rare. The fortunate possessor of one of these rare forms, well preserved in formalin, can ill afford to mar its beauty by even partial dissection. Radiography will enable him, without any injury to the entire organism, to determine the nature and relations of its skeletal parts. As the author points out, the figured radiograms afford considerable help in distinguishing between two closely allied species, *Asterias rubens* and *Murrayi*. The radiogram of *Astropecten irregularis*, with its markedly lateral madreporite, is particularly good. In the letterpress to this work, which is issued as a supplement to the *Archives of the Roentgen Ray*, Dr. Wolfenden has given a clear account of the methods he employs, and a brief description of the genera and species he has submitted to the Röntgen ray. It only remains to add that the plates are produced in admirable style, to congratulate the author on a good piece of work, and to wish him success in its further developments.

**Elementary Physiology for Nurses.** By C. F. Marshall, M.D. Pp. 89. London: The Scientific Press, Limited. 1897.—If it is any advantage to nurses to have a smattering of physiology they could not do better than begin by reading this little book, which gives a brief but fairly clear outline of the main functions of the various organs and tissues. The diagrams are helpful, and the descriptions of the circulation, digestion, &c., are good, so that a mere beginner can get some idea of the subject without great difficulty. Those, however, who have had experience of the teaching of physiology realise how hard it is for the average student or nurse to get a real understanding of the subject from such condensed manuals.

**The Means by which the Temperature of the Body is maintained in Health and Disease, being the Croonian Lectures, 1897.** By W. Hale White, M.D. Pp. 77. London: J. & A. Churchill. Reprinted in 1897.—These lectures give an account of the author's own observations on pyrexia, a subject which is always of paramount importance and of interest both to the physiologist and the physician. After describing a new form of calorimeter he has to regret that no instrument has yet been devised which will give trustworthy results; he also remarks that the study of the *chemistry* of the heat-production of the body is so beset with difficulties that not very much work has been done in this direction. The three main conclusions which he deduces from a discussion of the problem of the variations in the production and loss of heat in pyrexia are as follows:—(1) In man at least pyrexia is not produced by the same method in all fevers: sometimes the rise of temperature is due to an increased production of heat, and sometimes to a diminution of the loss. (2) In the present state of our knowledge animal calorimetry is very difficult, and the results obtained from calorimeters must be received very guardedly, especially when they are applied to man. (3) In some forms of pyrexia in which the production of heat is increased, the metabolism leading to this takes place in the proteid tissues of the body, and probably the metabolic processes concerned in a pathological rise of temperature are different from those of health. An interesting question is discussed whether or not pyrexia is a protective mechanism, and, if so, whether it might be a good thing to administer to fevered patients drugs that would raise their temperature, inasmuch as there are pyretic drugs as well as antipyretics. We must confess to a decided preference for the antipyretics, which do not appear to receive all the credit which their merits deserve.

**Air, Food, and Exercises.** By A. RABAGLIATI, M.D. Pp. xvi., 220. London: Baillière, Tindall & Cox. [N.D.]—We suppose Dr. Rabagliati writes in a serious vein, and accordingly we feel bound to criticise his work in a similar spirit, but we must confess that as we read through the pages the conclusion was forced upon us that the author was attempting a joke under the cloak of a semi-scientific publication. There is, however, a certain amount of pleasure and excitement in reading the theories of a man who attributes all diseases to a common cause. One wonders what disease will next be worked in, and in what way; how he will extricate himself out of some pathological slough, and come up smiling, and prove that he got into it only from his arguments. In a most ingenious way, starting from one disease, which the author declares to be due to excessive ingestion of amylaceous and saccharine food, he conclusively—to his mind but not to ours—proves that such remotely connected diseases as bronchitis, cancer, abscess of the liver, cerebral hemorrhage, and carbuncle are all due to the same cause. This is certainly a triumph of scientific reasoning of the authorship of which no one will try and rob Dr. Rabagliati. In spite of this balderdash, the book contains some smart and clever sayings, but being mixed up with a lot of trash these will probably not receive the attention they merit.

**Nervous Affections of the Hand, and other Clinical Studies.** By GEORGE VIVIAN POORE, M.D. Pp. 308. London: Smith, Elder, & Co. 1897.—Dr. Poore here brings together several of his lectures and papers which have been previously published. The most important of them is the Bradshaw lecture on the hand, which he delivered so long ago as 1881. Although this does not contain anything with which we are not familiar, the arrangement of the various points is excellent. The papers on "Professional neuroses" are exceedingly interesting, and include notices of writer's cramp, tailor's cramp, goldbeater's cramp, hammerman's cramp, the conditions which interfere with piano-playing, and sawyer's cramp. These are all well described. It seems almost a pity that these chapters should have been mixed up with other subjects of a different nature. The author's remarks on the deep reflexes in spinal myelitis are good, as are also those on various forms of poisoning, one of which, auto-intoxication, is interesting, as here insensibility may suddenly supervene on extreme muscular exertion. The other essays in the book are all good of their kind. There is something to be learned from them all. They range from gout following influenza, the relation of albuminuria to life-assurance, congenital and chronic heart disease, to the treatment of pulmonary tuberculosis, tumour of the lung, the sulphur waters of the Pyrenees, and laryngeal spasm.

**Spinal Caries.** By Noble Smith. Second Edition. Pp. vi., 153. London: Smith, Elder & Co. 1897.—We notice very few alterations or additions in this book, which is mainly a reprint of the former edition. A short appendix is added, in which the author in a few disparaging remarks dismisses the method recently introduced by Dr. Calot, of forced reduction of the deformity of caries under chloroform.

1. **Review of the New British Pharmacopœia, 1898.** Pp. 61. Nottingham: Boot's Pure Drug Co., Ld.—2. **The British Pharmacopœia, 1898: Alterations, Omissions, and Additions, [&c.]** Sixth Edition. Pp. 48. London: *Pharmaceutical Journal* Office. 1898.—3. **Pocket Notes on the New British Pharmacopœia, 1898.** Pp. 123. Bristol: Ferris & Company.—4. **A Synopsis of the British Pharmacopœia, 1898.** Compiled by H. Wippell Gadd. Pp. 183. Exeter: Evans, Gadd & Co. 1898.—Of the various guides to the new Pharmacopœia we have received the above examples. A brief notice of them must suffice. No. 1, reprinted from *The Chemist and Druggist*, is too lengthy and too largely pharmaceutical to be well adapted for use by medical men. The same, to a large extent, may be said of No. 2, although less space is assigned to pharmaceutical detail, and we should think the pamphlet would be valuable to students going up for examination in pharmacology and medicine. No. 3, issued by a local firm, appeals strictly to practitioners, and is very compact and concise; it assumes a knowledge of the old edition of the B. P., presenting information only respecting the alterations and additions and changes in nomenclature incorporated in the new B. P. We gladly commend the book. No. 4 has much also to recommend it: in form it is still more compact; it gives a practically complete synopsis of the 1898 B.P., and gives also what that work does not, as it ought to have done, the doses in metric as well in imperial measure. There are also tables of weights and measures, and other useful information, and it is a very valuable little book.

**The Practice of Massage: its Physiological Effects and Therapeutic Uses.** By A. Symons Eccles, M.B. Second Edition. Pp. 374. London: Baillière, Tindall & Cox. 1898.—This text book on the much abused subject of massage may be looked upon as a scientific exposition of the practice by one who is a complete master both of its theory and applications, *i.e.*, by "one who is at the same time a practitioner of medicine and a practical masseur." This combination rarely exists, the physician or surgeon seldom becomes an expert masseur, and the masseur no matter how expert is often lacking in scientific knowledge. Too much of what is called massage is mere unscientific rubbing of different varieties, and is often calculated to be more for the financial benefit of the operator rather than for the therapeutic advantage of the patient. The author "has a high appreciation of massage as a remedial agent of special effectiveness that comparatively few know or recognise at present; but if it is to take this place the manipulations in difficult cases must be practised by the doctor himself, not by any necessarily less educated person." He considers that in many cases the ordinary application of the remedy may well and rightly be confided to skilful manipulators acting under the supervision of medical practitioners, but that in other conditions, more especially in diseases of the gastro-intestinal tract, automatic massage by unskilled lay hands may be fraught with disaster. The main object of the book is to foster a wider acquaintance and a closer intimacy with the uses and limitation of massage amongst medical practitioners, and the fact that a second edition has been so soon required is good evidence that the book must be attaining this object.

**The Oxygen Treatment for Wounds, Ulcers, Burns, Scalds, Lupus, and Diseases of the Nose, Eye, and Ear.** By George Stoker. Pp. 39. London: Baillière, Tindall & Cox. 1897.—Oxygen is evidently Dr. Stoker's hobby; he looks upon it as a panacea for all evils. In the treatment of ulcers with it he claims rapid relief from pain, absence of smell, avoidance of exuberant granulation, very rapid healing, and a resultant scar not composed of ordinary cicatricial tissue. We must admit, on looking over some of his cases at the Oxygen Home, that we were not convinced on all these points, and that some cases that had been under treatment for many weeks were not more advanced in healing than one might reasonably have expected on ordinary surgical treatment, and if to this we might add Thiersch's grafting we think we could give oxygen points. Dr. Stoker observes that under the oxygen treatment as under ordinary dressings many chronic ulcers having rapidly healed to a considerable extent remain stationary just short of complete repair. The one advantage of the method is the avoidance of any irritating dressing in actual contact with the sore.

**Economics, Anæsthetics, and Antiseptics in the Practice of Midwifery.** By HAYDN BROWN. Pp. 103. London: J. & A. Churchill. 1897.—The introductory part of this work calls for no comment, it consists of truisms that we are all prepared to accept, in fact we have always laid it down that the doctor and the nurse may be two great dangers to the lying-in woman, and the success of their work depends in a great measure on their proper appreciation of their duties, as laid down by all modern writers on obstetrics and teachers of that branch of medicine. But we cannot agree with all the statements in chap. 2. For instance, in reference to the trouble of parturition, it is not a fact that "every married woman has to go through it." Again, at the first interview of patient and doctor a more soothing mode of handling the prospective mother is desirable than the author's recommendation of eliciting at that time the history of hysteria or epilepsy. Chap. 4 contains some startling statements: we are told it is necessary to pass the hand through the vagina in order to turn. In most cases all that is required is to pass a finger and turn by the bi-polar method. We agree with the author in considering there is great abuse in the use of instruments, but we do not agree with him in condemning axis-traction in suitable cases. We have succeeded with axis-traction forceps after failing with ordinary long forceps. In the routine questions to be asked after confinement the very important one concerning the condition of the bladder is omitted. We consider the author allows his patient to be up too soon. The chapter on nurses and midwives is a distinct proof of the necessity for legislation, as the ignorance here detailed of their work shows the necessity for their education and registration. The chapter on antiseptics is good, and we consider that Dr. Brown is right in preferring perchloride of mercury to any other disinfectant for the hands and instruments, but as a vaginal disinfectant after labour the use of iodoform pessaries is all sufficient.

**The Midwives' Pocket Book.** By HONNOR MORTEN. Pp. 93. London: The Scientific Press, Limited. 1897.—The great merit of this work is that it does not err in the direction of over confidence, simple rules for determining the approach of danger are laid down with their corollary of "send for the doctor," while much useful information as to training schools, etc., is given. If the book reaches a second edition the spelling of "bi-pareital" (p. 46) should be corrected. The bi-temporal diameter of the child's head is under-estimated at 2½ inches and the description of the perineum as "a wedge-shaped tissue" is not very informing.

**Lawson Tait's Perineal Operations, and an Essay on Curettage of the Uterus.** By W. J. STEWART MCKAY, M.B. London: Baillière, Tindall & Cox. 1897.—Judged as an example of mere book-making, this publication is about the worst we have seen.

"Perineal Operations."—the lettering on the back—becomes on the side of the cover, "Lawson Tait's Perineal Operations," to finally blossom out on the title page into the form as given at the head of this notice. Inside there is about a page of preface by Mr. Tait, and then, after about two pages of introduction by the author, the dissertation on "Tait's Perineorrhaphy" occupies less than 16 pages, and is followed by 43 pages on "Curettage of the Uterus," and 40 pages of publishers' advertisements. Lawson Tait's operation, however, is very well described and the diagrams are real aids to the understanding of its various steps. Curettage of the uterus is an operation not to be undertaken so frequently or for so many diseases as recommended by the author, for according to him almost every operation that the uterus has to undergo requires to be supplemented by curettage. In this respect we consider this book to be highly dangerous. The whole essay reflects the evil tendency of some gynæcologists not to give nature a chance. Although in suitable cases and by experienced men curettage is a valuable proceeding, the ordinary doctor had better be content to leave it alone.

**The Science and Art of Adjustment between the Producing and Reflecting Vocal Apparatus.** By Paul Mahlendorff. Pp. 40. London: Marriott & Williams. 1897.—This pamphlet contains nothing of the least value to learners of the art of singing.

**Hygiene and Public Health.** By Louis C. Parkes, M.D. Fifth Edition. Pp. xix., 551. London: H. K. Lewis. 1897.—We are glad to see that this very useful Students' Handbook has reached its fifth edition. It has been revised, and to a slight extent enlarged in order to keep abreast of advancing knowledge, and in its present form is both clear and trustworthy.

**The Sanitary Inspector's Handbook.** By Albert Taylor. Second Edition. Pp. xvi., 332. London: H. K. Lewis. 1897.—This is a really practical book, and excellently designed to help the Sanitary Inspector to a clear understanding of his multifarious duties. Approved forms for registers, pocket-books, and notices are supplied, and judicious hints are given as to the conduct of work, and the Inspector's dealing with the public. The medical notes are sufficiently full to be helpful, without unnecessary detail; from them the intelligent inspector could readily gather when it is necessary to call in the aid of the medical officer.

**A Manual of Hygiene for Students and Nurses.** By John Glaister, M.D. Pp. xv., 295. London: The Scientific Press, Limited. 1897.—We have in this a popular, but carefully written handbook, containing the more important facts of modern hygiene. Some excellent suggestions as to rural Isolation Hospitals are given, with sketch plans.

**Memoria de los Trabajos ejecutados por el Consejo Superior de Salubridad en el Año de 1895.** Pp. x., 158. Mexico: Imprenta del Gobierno en el ex-Arzobispado. 1896.—The city of Mexico, situated in the valley of the same name, is the largest of the Republic, and contains a population of about 350,000 inhabitants, who are chiefly Mexicans of Spanish descent, Indians, and a few hundred natives of the United States and Great Britain. The streets are generally wide, but badly-paved and ill-kept. One of the most remarkable features of the city is the number of splendid residences, built round "patios," or courtyards, in which are bright and sweet-smelling flowers, tall palms and fountains. But the most beautiful city in the world can be spoiled by imperfect drainage, and this was pre-eminently the case here; for, prior to 1895, the sanitary condition was in a deplorable state: as there was little or no sewerage, and cesspools and public dunghills were the order of the day, while in the streets were deposited all kinds of filth and garbage. The consequence, naturally, was that in 1893 the mortality of the city reached the high figure of 20,428, or 58.3 per thousand, and, of these deaths, typhus fever was responsible for 322, typhoid for 4, smallpox for 50, scarlatina for 26, and diphtheria for 12, the predominance of typhus over typhoid being remarkable. At last the authorities bestirred themselves in the matter, and, mapping out the town into eight districts, they sent an inspector, accompanied by a medical man, into each, and, acting on their reports, they borrowed a large sum of money in Europe, and constructed sewage works on the latest principles and on an extended scale, with the gratifying result that the city is now one of the healthiest in America.

**B. Bradshaw's Dictionary of Bathing Places and Climatic Health Resorts.** London: Kegan Paul, Trench, Trübner & Co., Ltd. 1898.—The new edition of this useful book contains 362 pages of matter, comprising much information on bathing places, climatic health-resorts, mineral waters, sea baths, and hydropathic establishments. It also specifies some doctors and some hotels that can be recommended with confidence, and gives numerous maps and plans. It is, perhaps, too much to expect that the names of seven (!) Clifton doctors should be accurate, or that there should be any mention of the new Grand Spa Hydro.

**Bath as a Health Resort.** Pp. 59. Published by the Bath Corporation. [N.D.]—In bringing this publication before our readers we cannot do better than quote its preface, which says: "The object of this Sketch-book, which is issued by the Baths Committee of the Bath Corporation, is to make more widely known not only the medicinal virtues of the Hot Springs and the completeness of the Bathing Establishments, but to convey some idea of the residential attractions and advantages of the City, and the historical interest attaching to a locality in which the remarkable remains of the Roman Occupation are so wonderfully preserved."

**Fevers and Infectious Diseases: their Nursing and Practical Management.** By WILLIAM HARDING, M.D. Pp. 88. London: The Scientific Press, Limited. 1897.—This little book will prove invaluable to those engaged in the work of nursing cases of infectious disease. The nursing information given in it is eminently practical, but the disinfection of the sick-room after the patient's removal is not a matter which should be left to the nurse. It is to be regretted that the space devoted to this subject was not employed in giving more minute details as to the treatment of infectious dejecta, and to the consideration of measures to secure the personal cleanliness and freedom from infection of the sick attendant. The too frequent occurrence of enteric fever amongst those nursing cases of this disease points to the fact that nurses, either from ignorance or carelessness, often perform the duties of personal and general disinfection in the most imperfect manner.

**Transactions of the Clinical Society of London.** Volume XXX. London: Longmans, Green, and Co. 1897.—**An Index to the Transactions of the Clinical Society of London, Vols. I.—XXX.** London: Longmans, Green, and Co. 1898.—The present volume is a collection of interesting papers, mostly on surgical cases, many of which, no doubt, gave rise to much important discussion. The book has a carefully-made index, and is in every way equal to the usual high standard of the previous volumes. Upon the completion of this volume the Society has issued an index of the whole series. In this instance the benefactor to his generation and to posterity is Dr. Archibald E. Garrod, upon whom the entire labour of preparing it has fallen. We render our cordial thanks to him for it.

**Transactions of the Medical Society of London.** Vol. XX. London: Harrison and Sons. 1897.—The President, Mr. Reginald Harrison, discusses the treatment of some forms of albuminuria by reni-puncture. The arguments are based on clinical observations, and the treatment is suggested for acute nephritis with much tension in the kidney and for chronic nephritis which is not improving otherwise. Mr. John D. Malcolm describes twenty-six cases in which abdominal section was performed a second time. Three cases consist of growths in the second ovary after unilateral oöphorectomy. The remainder comprise various complications and accidents which have arisen. In an article upon the best methods of removing large calculi from the bladder Mr. P. Freyer says that he has now almost completely abandoned every other method of treatment of stone in favour of Bigelow's litholapaxy. He has only done seven cutting operations in his last three hundred cases, and in a series of one hundred and six cases of litholapaxy he had only one death. The term, a large stone, is relative to the age of the patient and the experience of the operator. Dr. Haig gives reasons why he regards the uric acid diathesis as a myth. Mr. Mayo Robson relates cases of appendicitis with so-called general peritonitis which recovered after operation. Mr. Bland Sutton reviews the question of wandering spleens. Dr. Samuel West, in an able paper, speaks favourably of the prognosis of pneumothorax. The Lettsomian lectures by Dr. de Havilland Hall on diseases of the nose and throat in relation to general medicine comprise a very thorough exposition of the subject well worth reading. Mr. Hurry Fenwick's paper on the value of the cystoscope in reducing the mortality from nephrectomy has been already published. Mr. Lockwood describes a method of dovetailing the bones in excision of the knee. Mr. Armstrong speaks of the value of exclusive diet of red meat in chronic gout. Mr. Swinford Edwards gives details of fourteen cases of Kraske's operation for cancer of the rectum. There are also several other papers in the volume, as well as records of cases. The early record of the Medical Society is well presented in the annual oration, which this year was delivered by Mr. Edmund Owen. The Society possesses an excellent library, especially of older medical works; but Lettsom, who laid its foundation and was its first guardian, can scarcely think that its needs are at all adequately supplied by the addition during last year of fifty-nine volumes.

**Medico-Chirurgical Transactions.** Vol. LXXX. London: Longmans, Green, and Co. 1897.—The address of the president, Dr. William Howship Dickinson, contains obituary notices of a long list of deceased Fellows, including such names as Russell Reynolds, George Johnson, John Eric Erichsen, George Murray Humphry, Langdon-Down, Thomas Spencer Wells, George Harley, and Edward Ballard. The interesting account of so many medical and surgical giants gives the volume an exceptional value. The following 415 pages contain the usual mixture of communications read at various meetings throughout the year: among these we may especially notice a case of recovery after operation on perforated typhoid ulcer by Mr. Bowlby; also a case of laparotomy for simulated typhoid perforation in which no lesion was found, and recovery was uneventful. A paper on the presence of typhoid bacilli in the urine suggests the necessity for antiseptic treatment of the urine in all cases of typhoid fever.

**Transactions of the Association of American Physicians.** Vol. XII. Philadelphia: Printed for the Association. 1897.—The annual meeting of this Association of one hundred and twenty-five physicians of eminence is quite a noteworthy event, and the volume recording the proceedings of the meeting, held at Washington in 1897, is before us. The president, Dr. J. M. Da Costa, remarks that "with everyone of us there is a mission to assist the general advance"; and this is the tone which pervades the volume throughout its five hundred pages, where we have the latest experiences of the serum test for typhoid fever, cases of gall-bladder infection in typhoid, the hepatic complications of typhoid fever, the effect of the cold bath treatment in favouring elimination of urea in typhoid, and many other topics giving the experience of many of the best observers of our time. The use of the "fluoroscope" in the diagnosis of diseases of the heart and lungs shows that the use of the Röntgen rays need not be limited to the surgeon or to pins and buttons.

**Transactions of the Michigan State Medical Society.** Vol. XXI. Grand Rapids: Published by the Society. 1897.—Among the papers in this volume we may refer to Dr. J. H. Kellogg's article on his method of shortening the round ligaments, which he has employed in 591 cases. In his last 200 he records less than 2 per cent. of failures, though a certain proportion of the patients still complain of the backache and other pains after the operation. These he regards as due in part to other troubles complicating retroversion. Dr. Roswell Park sums up our present knowledge of the nature and cause of cancer. There is also a noteworthy group of papers by various authors on cancer of the stomach. Drs. T. H. Walker and J. H. Kellogg write on the diagnosis, while Drs. McGraw and Wyman treat of the surgical measures indicated. Dr. McGraw, while pointing out the high mortality of operations on the stomach, shows that a few operators have obtained highly successful results with low mortality, and puts in a plea for early diagnosis and complete excision of the growth in pyloric cancer. A few surgeons have had even nine or ten consecutive cases each of recoveries. In advanced cases, if the patient is otherwise in good health, he considers that gastro-enterostomy is not a very dangerous proceeding, while the relief from suffering which it gives is great and striking. The numerous operations which have been performed on moribund patients have caused a wrong idea of the danger of the operation. In short, he is most hopeful as to the results of surgical treatment in future.

**Transactions of the New Hampshire Medical Society.** Concord: Ira C Evans. 1897.—Perhaps the most noteworthy paper in this volume is that by the late Judge Foster on "Medical Expert Testimony." The courts of New Hampshire have abandoned the attempt to define "unsound mind" legally, and no longer regard delusions, or a knowledge of right and wrong, as tests; but the sole enquiry is—Was the act the product of mental disease? The president, Dr. Abel P. Richardson, took Old Age as the subject of his address, and dealt with it in a practical way. He dismissed the claims to longevity such as those put forward for Parr and others, and considers that no one in England or the United States has ever reached the age of 110.

Papers are also given on neurasthenia; on acute abdominal affections, with special reference to the time for calling in the surgeon; the application of the X-rays; degeneration of eyes; and on a few other subjects, several of which resemble clinical lectures of a post-graduate course.

**Proceedings of the Philadelphia County Medical Society.** Vol. XVII. Philadelphia: Printed for the Society. 1896.—This volume, of 254 double-column pages, is a record of much good work, done by good observers. A paper by Dr. Anders on typhoid fever as a complication and a sequel of influenza may be of especial interest in this neighbourhood; but no bacteriological observations were undertaken, and none were required to verify the diagnosis of typhoid fever: it is difficult to see on what ground the cases were considered to be influenza at all, and why they should not be accepted as simple cases of enteric fever of slow and irregular development. There is, however, no *a priori* reason why a patient who is incubating typhoid fever may not have an intercurrent attack of passing febrile influenza, but there appears to be no absolute connection between them, and we think it most undesirable that they should be mentally associated, lest the diagnosis of the minor may disguise the onset of the major malady. Dr. Joseph Price's cases of typhoid perforation, three of them, all terminating in successful recovery, give encouragement to persevere in a practice which is commonly less successful: he pleads for early interference in these cases, as delay is fatal, and adds that "early diagnosis, early operation, painstaking, rapid work will save many lives." A paper by Dr. Stengel on the treatment of pernicious anæmia emphasises the utility of arsenic; but he finds that bone marrow has not given very satisfactory results. We think that iron is a drug which has been abundantly proved to be harmful in this form of anæmia, and that it should be reserved for the simple chlorotic forms. The practice of giving iron and arsenic together as a routine practice in anæmia is not grounded on scientific knowledge, and on the evidence before us should be reserved for those doubtful cases where a definite diagnosis does not seem to be possible and where neither of the two drugs administered separately gives good results.

**Transactions of the American Pediatric Society.** Vol. VIII. Reprinted from *The Archives of Pediatrics*. 1896.—Of the numerous communications published in this volume perhaps the most valuable is an exhaustive report on cases of diphtheria treated by antitoxin, to which reference was made in the *Journal* for December, 1896. There are also two interesting papers giving the results of careful work on puncture of the subarachnoid space in cases of meningitis, and another on "The Pasteurization of Milk." The remaining papers are mainly the reports of cases of special interest. Of the papers on "Lumbar Puncture of the Subarachnoid Space," one is by Dr. Wentworth, the work emanating from the pathological laboratory of the Harvard Medical School, the other by Dr. Jennings, of Detroit. The latter gives the result of the examination of twenty-one cases, the former of twenty-nine. Dr. Wentworth remarks that the majority of practitioners, of course, are not in a position to make cultures or inoculation experiments, and that the staining for bacilli and other organisms is tedious. He thinks, however, that a good deal can be learnt from the character of the cerebro-spinal fluid. In the normal state it is perfectly clear and deposits no cells, whereas in cases of meningitis it is invariably cloudy. This cloudiness is caused by cells, which examined microscopically appear to be generally small round cells with a single nucleus, whereas in the purulent forms of meningitis polynuclear leucocytes are more numerous. In his paper on "The Pasteurization of Milk," Dr. Freeman considers that so high a temperature as 75° C. need not be adopted; that between 65° and 70° C. is sufficient to destroy almost all the micro-organisms present in milk, including the tubercle, typhoid, and diphtheria bacilli. At this lower temperature the taste is not altered and the chemical changes to be avoided are not set up.

**Transactions of the American Laryngological Association.** New York: D. Appleton and Company. 1897.—These transactions may be taken as very fairly representative of American laryngology, and afford clear evidence of the scientific character of the work that is being done in this speciality in the New Hemisphere. A paper by Dr. Jonathan Wright especially attracts our attention, not because it is intrinsically of greater excellence than many of the others, but because it deals with the much-debated question of tuberculous infection through the tonsils. "The investigations of Baumgarten, Sims Woodhead, Krückmann, and others prove pretty conclusively that the bacilli do get through the epithelium of the throat and into the cervical lymphatics in tuberculous subjects and in animals fed on tuberculous food. Were it not for clinical experience, therefore, and such investigations as those of Dr. Hodenpyl, it would seem extremely probable that the lymphoid tissues of the throat should contain tubercle. Dr. Hodenpyl examined about two hundred sections for bacilli and found none, nor anything like tubercle in tonsils." Dr. Wright therefore repeated Dieulafoy's experiments, and five instances of removal of enlarged faucial tonsils, and seven of post-nasal "adenoids" gave negative results for tubercle when inoculated in guinea-pigs. Yet another case of clinically apparent tubercle of the tonsil gave positive results, and afforded characteristic evidence of tubercle histologically. After examining the work of other observers on this question, Dr. Wright is inclined, both from clinical and pathological evidence, to agree with Dr. Hodenpyl, when he says that tuberculous amygdalitis is a rare affection, and that the tonsils are rarely the seat of primary inoculation. Dr. Wright also gives an excellent coloured reproduction of a stained microscopical section, showing the infiltration of the epithelium of the larynx by tubercle bacilli, and thus proving the possibility of direct infection by this method, a point of very great pathological import.

**The Edinburgh Medical Journal.** New Series. Vol. III. Edinburgh: Young J. Pentland. 1898.—This volume contains many papers of more than ephemeral interest and importance. Those by Dr. Frederick T. Roberts on the systematic physical examination of the chest are of special value. He points out that "while physical examination still holds its own, on the whole, in theory, it cannot be questioned that in practice, as it is carried out in a large proportion of cases, it might as well be omitted altogether," and he pleads for a plan of procedure which aims "to fix attention upon the several *objects* or *purposes* for which physical examination is intended," rather than a study of the physical signs under each *method*.

**Annales de la Société Belge de Chirurgie.** Sixième Année, Bruxelles: Henri Lamertin. 1898.—We are glad to have this publication on our exchange-list. The opening numbers of the current volume contain articles on ligature of the external carotid, the surgery of the liver and kidney and other subjects, contributed by eminent Continental surgeons.

Mr. Young J. Pentland informs us that he is about to issue several new works, amongst which we notice *A Text-Book of Medicine*, by British Teachers; *Contributions to Clinical Medicine*, by Dr. McCall Anderson; *Diseases of the Heart and Aorta*, by Dr. G. A. Gibson; *Lectures on Giddiness and on Hysteria in the Male*, by Sir Grainger Stewart; *The Principles of Treatment*, by Dr. Mitchell Bruce; *The Radical Cure of Hernia, Hydrocele, and Varicocele* by Mr. C. B. Lockwood; and *A Manual of Midwifery*, by Dr. Milne Murray. A new edition of Dr. Osler's *Medicine* is also announced, and also the second volume of the *Text-Book of Physiology* edited by Prof. Schäfer. The excellent *Journal of Pathology and Bacteriology* is about to enter on its fifth volume.

Mr. W. B. Saunders tells us that he has several important works almost ready for issue. Among the new books are several Text-Books: *Pathology*, by Dr. Stengel; *Obstetrics*, by Dr. Barton Cooke Hirst; *Diseases of the Eye, Ear, Nose, and Throat*, edited by Drs. de Schweinitz and Randall. New editions are promised of Dr. DaCosta's *Modern Surgery*, of Dr. McFarland's *Pathogenic Bacteria*, and of the well-known *American Text-Books of Diseases of Children* and *Gynecology*. Mr. Saunders will also shortly publish revised editions of Dr. Griffith's *Care of the Baby*, Dr. Butler's *Materia Medica and Therapeutics*, and of the English version of Vierordt's *Medical Diagnosis*. This notice would be incomplete without a special word of praise for Mr. Saunders's share in giving to English readers a series of the "Lehmann Medicinische Handatlanten." Five of these are now ready, and eight are in active preparation. Many who are acquainted with this series in the original will appreciate the provision of the volumes in English at the moderate price at which they are issued.

All the books published by Mr. Saunders can be obtained in England through the Rebman Publishing Company.

### Notes on Preparations for the Sick.

